# Design of a Fano-resonance-enhanced dielectric grating for ultralow-filling-factor superconducting nanowire single-photon detector

**DOI:** 10.1038/s41598-026-58781-8

**Published:** 2026-06-20

**Authors:** Fan Zheng, Kejing Wei, Xiuli Huang, Junxia Zhao, Nan Chen, Jing Qiang, Lili Cheng, Ang Li

**Affiliations:** School of Intelligent Control, Nanjing University of Science and Technology ZiJin College, Nanjing, 210023 P. R. China

**Keywords:** Materials science, Nanoscience and technology, Optics and photonics, Physics

## Abstract

Superconducting nanowire single photon detectors (SNSPDs) exhibit excellent performance in the near-infrared band, but their application range is limited by the detection efficiency and detection speed. In this paper, we propose a high-efficient and ultralow-filling-factor design scheme based on Fano resonance which is excited by a one-dimensional silicon dielectric grating. This design can reduce the kinetic inductance of the nanowires while maintaining the active sensing area of SNSPDs, which is beneficial for improving the recovery time of the detector. Meanwhile, the absorption efficiency of the nanowires can be enhanced by the Fano resonance. Taking λ = 1550 nm as an example, numerical simulations are performed using commercial simulation software based on the finite-difference time-domain method. Under idealized structural conditions, the absorption efficiency of the Niobium Nitride (NbN) superconducting nanowires can exceed 98% when the filling factor of the nanowire is only 10%.

**Supplementary Information:**

The online version contains supplementary material available at https://doi.org/10.1038/s41598-026-58781-8.

## Introduction

Superconducting nanowire single-photon detectors (SNSPDs) have gradually become important detection devices in quantum communication, precision spectroscopy, light detection and ranging (LiDAR) and other fields owing to their excellent performance, such as high detection efficiency, low dark count rate, low timing jitter and fast response speed^1–17^. In the fields of LiDAR and imaging, SNSPDs are usually required to possess both high system detection efficiency (SDE) and high detection speed simultaneously^18–20^.

In these applications, large-aperture fibers are commonly packaged to collect as many photons as possible. Under these conditions, the active sensing area of SNSPDs must be enlarged to ensure efficient photon coupling into the detector^21^. However, increasing the sensing area simultaneously increases the total nanowire length, which will result in larger kinetic inductance and longer recovery time^21,24^. The performance of the SNSPDs is limited by the physical properties of the nanowires themselves. To achieve fast detection, nanowires are usually fabricated into an ultralow filling factor structure to reduce the kinetic inductance, shorten the recovery time and improve the detection speed^21–23^. However, a lower filling factor will result in a smaller coverage area of the nanowires. Although the local electric field intensity around the nanowires is slightly enhanced, a large number of incident photons cannot interact effectively with the nanowires, which will reduce the photon absorption probability^21,24,25^. This forms a technical bottleneck that makes it difficult to balance efficiency and speed.

To address this challenge, Li Guanhai et al.^26^ proposed a design scheme using metallic metamaterials to excite Fano resonance, thereby achieving both high detection efficiency and high detection speed. Nevertheless, metallic materials exhibit significant Ohmic loss in the infrared band, leading to energy dissipation of incident light. You Xiao et al.^21^ successfully resolved this issue by utilizing a 2D photonic crystal, achieving an absorption efficiency of 90% with a nanowire filling factor of ~ 12%. Although this result is impressive, the complex structure of the 2D photonic crystal poses certain challenges for device fabrication.

Therefore, this paper proposes a design scheme based on Fano resonance excited by a one-dimensional silicon dielectric grating^27,28^. This scheme achieves an absorption efficiency of more than 98% under the ultralow nanowire filling factor of 10%. Meanwhile, the adoption of the one-dimensional silicon dielectric grating structure not only reduces the device fabrication difficulty but also avoids metallic Ohmic loss. This design provides a potential research pathway for the development of high-efficiency and high-speed SNSPDs.

## Results

### Theoretical analysis

It is well-known that the system efficiency *η*_system_ of the SNSPDs can be expressed as follows^9,29^:

1 $$\eta _{{{\mathrm{system}}}} ~ = ~\eta _{{{\mathrm{coupling}}}} ~ \times ~\eta _{{{\mathrm{absorption}}}} ~ \times ~\eta _{{{\mathrm{registering}}}}$$

where *η*_coupling_ is the device coupling efficiency, *η*_absorption_ is the device absorption efficiency, and *η*_registering_ is the device intrinsic efficiency.

To improve coupling efficiency, several effective coupling packaging methods have been developed, including cryogenic nanopositioners and novel self-alignment techniques^5^, with losses as low as 1%^5,30,31^. The intrinsic efficiency is directly and closely dependent on the superconducting quality, geometric design, and fabrication precision of the superconducting nanowires^5^. In particular, the nanowire width, thickness, and geometric uniformity can influence hotspot formation, current crowding, and the probability of photon-triggered resistive transitions^32^. Based on the saturated plateau in SDE dependence of the bias current, the intrinsic efficiency can reach unity in SNSPDs made of various materials, including NbN, WSi, and MoSi^5,33–35^. Therefore, to improve the absorption efficiency of the nanowires is the critical factor for enhancing the system detection efficiency.

The superconducting nanowire of a SNSPD is normally fabricated in a meander pattern with overall dimensions of several micrometers. Since the meander nanowires are composed of periodically repeated segments with identical geometric parameters and spacing, adjacent nanowire sections exhibit similar local optical responses under normal optical incidence. Therefore, the overall meander-shaped structure can be approximated as a 1D periodic structure^24^. The absorption efficiency of such a periodic structure is as follows^36^:

2 $$\eta _{{{\mathrm{absorption}}}} = \frac{{\mathop \smallint \nolimits_{{ - p/2}}^{{p/2}} \mathop \smallint \nolimits_{0}^{t} \frac{1}{2}\mathrm{Im} [\varepsilon ]\left| E \right|^{2} dxdy}}{{\mathop \smallint \nolimits_{{ - p/2}}^{{p/2}} \frac{1}{2}\left( {\frac{{\varepsilon _{0} }}{{\mu _{0} }}} \right)^{{1/2}} \left| {E_{0} } \right|^{2} dx}}$$

where *E*_0_ denotes the incident electric field; *E* denotes the electric field inside the superconducting nanowire; *p* denotes the pitch of the unit cell; *ε* and *t* denote the permittivity and thickness of the superconducting nanowire, respectively; and *ε*_0_ and *µ*_0_ denote the permittivity and permeability of the background material, respectively. It is apparent from Eq. (2) that when the incident field *E*_0_ is given, the absorption efficiency is determined by the internal field *E*. Therefore, investigating the near-field features of *E* within the nanowire cross-section is of great importance for determining the origin of the absorption efficiency of SNSPDs.

### Design of a one-dimensional silicon dielectric grating

**Fig. 1:**
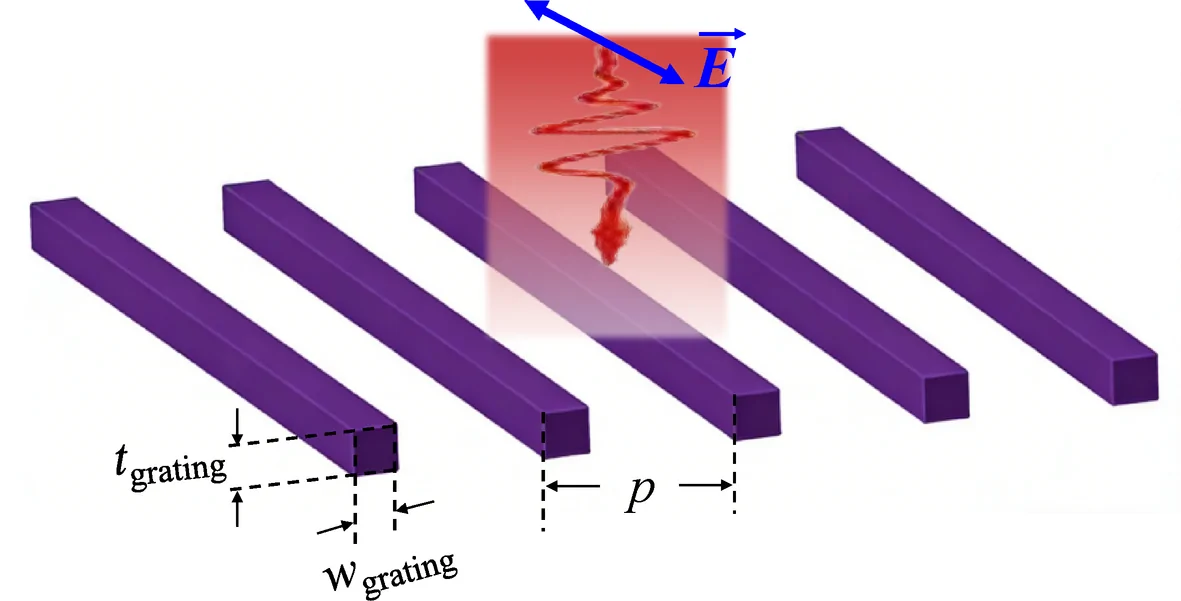
Schematic diagram of a one-dimensional silicon dielectric grating.

We start the SNSPD design by analyzing the electric field distribution of Fano resonance. Considering the difficulty of the fabrication, a one-dimensional silicon dielectric grating is selected to excite Fano resonance^37^, which is shown in Fig. 1. The thickness, width and period of the grating are defined as *t*_grating_, *w*_grating_ and *p*, respectively. Due to the one-dimensional periodicity of the grating, the simulation is performed on a 2D unit cell with periodic boundary conditions applied on the left and right boundaries, and perfectly matched layer boundaries at the top and bottom. The simulation is excited by a plane wave incident from vacuum, with its electric field polarized parallel to the grating. The incident electric field amplitude is set to 1 V/m, and the excitation wavelength ranges from 1200 nm to 1800 nm. The optical material used here is only silicon and the refractive index is 3.478(*n*_Si_=3.478)^38^ at 1550 nm.

**Fig. 2:**
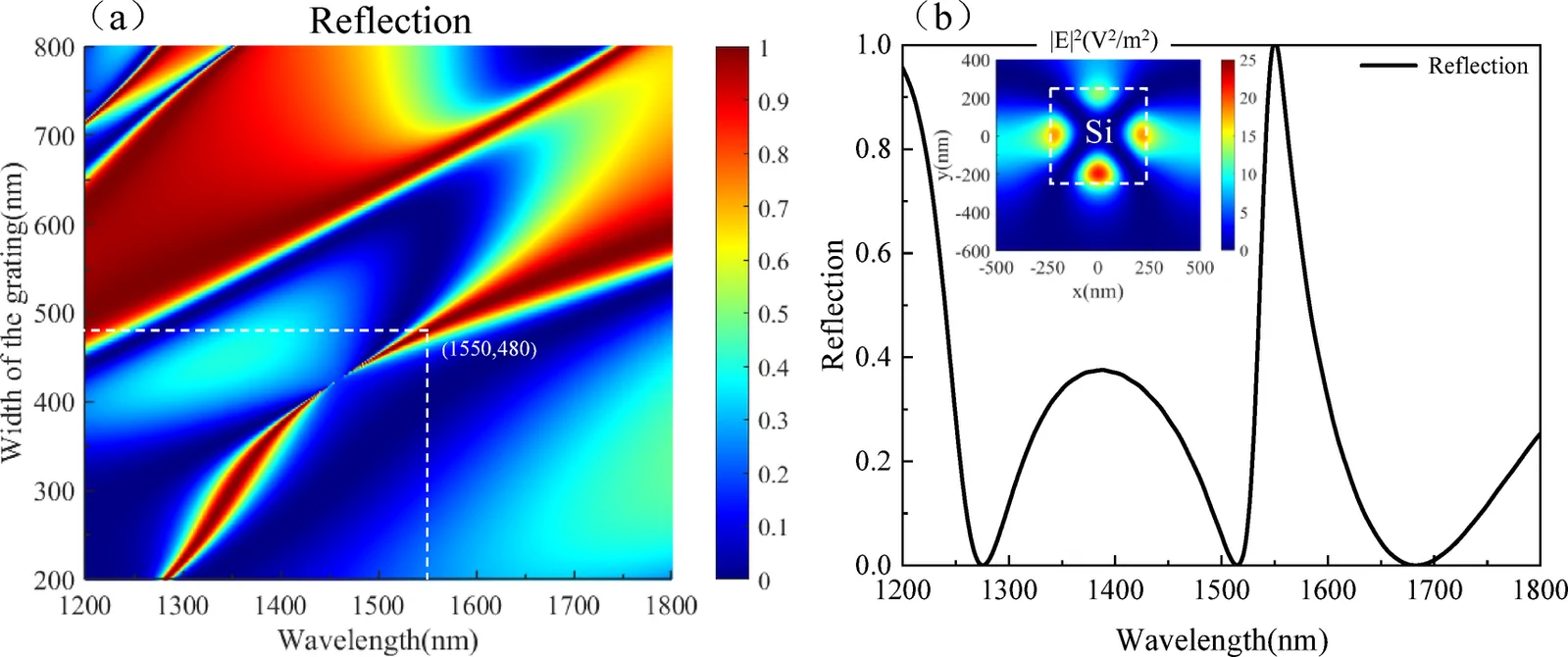
Calculated optical response of the one-dimensional silicon dielectric grating shown in Fig. 1. (**a**) Reflectance spectrum of the grating as a function of the grating width for the wavelength ranging from 1200 nm to 1800 nm. (**b**) Reflectance of the grating when *w*_grating_ = 480 nm, *h*_grating_=500 nm and *p*=1000 nm. The inset shows the distribution of the electric field intensity (|E|^2^) around the grating at Fano resonance.

To determine the structural parameters for Fano resonance excitation in the grating, the reflectance spectrum as a function of the grating width is calculated and presented in Fig. 2(a). In this simulation, commercial simulation software based on the finite-difference time-domain method was employed and the grating thickness and period are fixed at 500 nm (*h*_grating_=500 nm) and 1000 nm (*p* = 1000 nm), respectively. The grating width is varied from 200 nm to 800 nm. It can be found that the grating supports a pronounced Fano resonance at *w*_grating_=480 nm when the incident wavelength is 1550 nm. Under these structural parameters, the reflectance is nearly unity, which is shown in Fig. 2(b). Meanwhile, the electric field intensity distribution around the grating at the Fano resonance is calculated in the inset of Fig. 2(b). It is shown that the maximum electric field intensity is located directly below the grating, with a peak value of |E|^2^_max_ = 21.6V^2^/m^2^.

### Design of the SNSPD

**Fig. 3:**
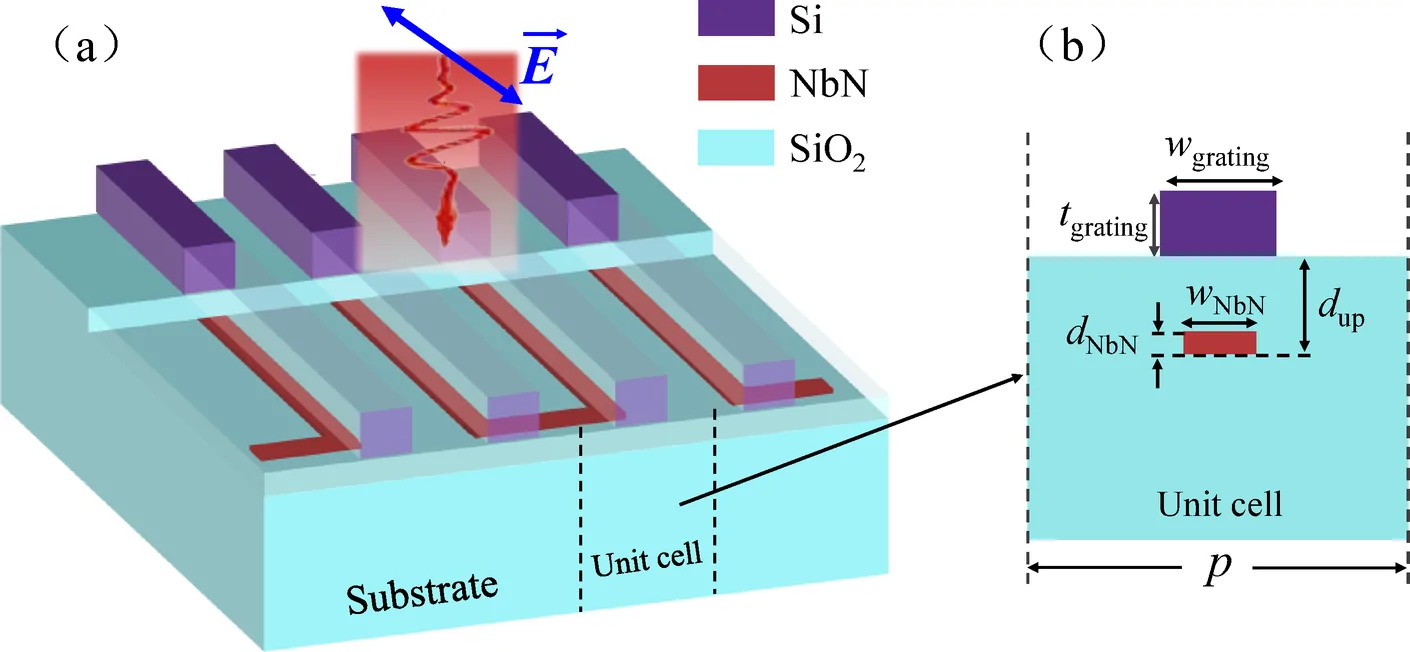
Schematic diagram of the SNSPD design with the one-dimensional silicon dielectric grating. (**a**) Three-dimensional schematic diagram of the SNSPD structure. (**b**) Two-dimensional cross-sectional schematic diagram of the unit cell.

Based on the above analysis, a design of the SNSPD is implemented and shown in Fig. 3. The device employs silica as a substrate, on which NbN nanowires are fabricated. The width and thickness of the nanowire are 100 nm (*w*_NbN_=100 nm) and 7 nm (*h*_NbN_=7 nm), respectively. A 50-nm-thick silica layer (*d*_up_=50 nm) is deposited on top of the nanowires to protect them from damage during the grating fabrication process. Finally, a one-dimensional silicon dielectric grating is fabricated on top of the structure to excite the Fano resonance.

Since the nearly periodic geometry of the NbN meander, a single unit cell was used in the simulation domain to represent the whole device. Considering the nanowires and the grating are all parallel to the electric field of the incident light, a 2D unit cell is employed for the simulation, which is consistent with the previous grating simulations. Periodic boundary conditions were imposed along the lateral direction, while perfectly matched layers were applied at the upper and lower boundaries to eliminate artificial reflections from the computational window. The incident light illuminates the structure from top to bottom, with the electric field polarized parallel to the grating. The period of the 2D unit cell is set to 1000 nm. To accurately resolve the strong field confinement and absorption behavior in nanoscale regions, a highly refined mesh of 1 nm was adopted inside the NbN nanowire region. Outside these critical near-field regions, a nonuniform mesh strategy was employed to improve computational efficiency, with the mesh size varying from 1 nm to 50 nm. The optical materials involved in the simulation include Si, SiO₂, and NbN, with their refractive indices at 1550 nm being *n*_Si_=3.478^38^, *n*_SiO2_=1.444^38^, and n_NbN_=5.23 + 5.82i^39^, respectively.

**Fig. 4:**
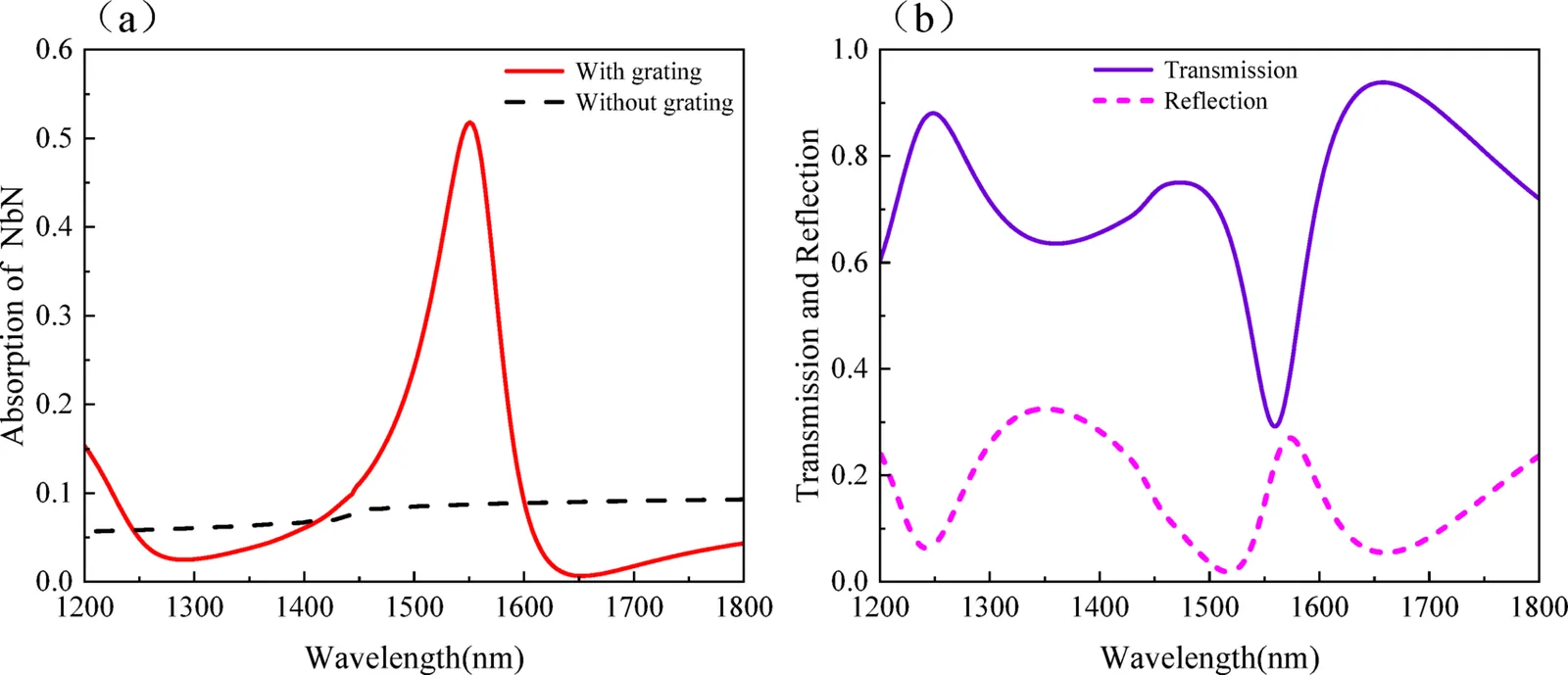
Optical response of the SNSPD structure without metallic mirror. (**a**) Absorption curves of NbN nanowires with and without the grating. (**b**) Transmission curve and reflection curve of the SNSPD structure.

During this simulation, considering the structural difference between the SNSPD and the original grating, minor adjustments to the width of the grating are required to ensure that the absorption peak is located at λ = 1550 nm. At this point, the structural parameters used in the simulation are as follows: *w*_grating_=475 nm, *h*_grating_=500 nm, *d*_up_=50 nm, *w*_NbN_=100 nm, *d*_NbN_=7 nm and *p*=1000 nm. To comparatively analyze the enhancement effect of Fano resonance on the absorption efficiency, the absorption curves of the SNSPD with and without the grating are calculated and shown in Fig. 4(a).

The simulation results show that the absorption efficiency of the nanowires is less than 10% when the device is without the grating. Compared with the previous results, the low absorption efficiency is primarily due to the ultralow filling factor of the NbN nanowires, which will reduce the coverage area of nanowires^24,25,36^. After the silicon dielectric grating is integrated into the device, the absorption efficiency of the nanowire is significantly enhanced to 51.8% at λ = 1550 nm. It is confirmed that the grating has a significant enhancement in absorption efficiency of nanowires when Fano resonance is excited. However, an absorption efficiency of approximately 50% cannot meet the requirements for high-efficiency detection in SNSPDs. In order to further enhance the absorption efficiency, we calculate the reflectance and transmittance of this SNSPD structure, as shown in Fig. 4(b). It can be seen that the transmittance is consistently higher than the reflectance, which indicates that a large portion of the energy transmits through the nanowires without being absorbed. Therefore, the design of SNSPD needs to be optimized to reduce the transmission.

**Fig. 5:**
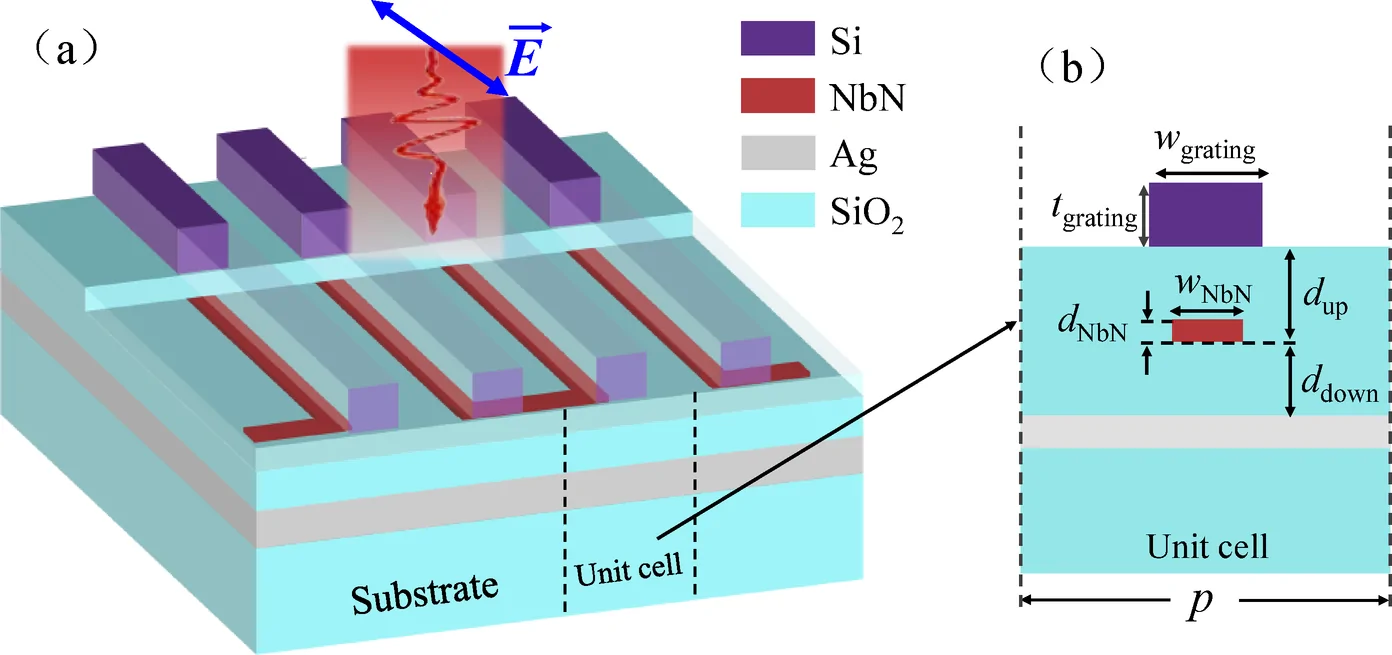
Schematic diagram of the SNSPD design with the grating and metallic mirror. (**a**) Three-dimensional schematic diagram of the SNSPD structure. (**b**) Two-dimensional cross-sectional schematic diagram of the unit cell.

To address this issue, the design of this SNSPD is improved and shown in Fig. 5. The device still adopts silica as the substrate, on which a 200-nm-thick silver layer is fabricated to act as a metallic mirror. The gap between the metallic mirror and the NbN nanowires is 150 nm (*d*_down_=150 nm), and the region between them is filled with silica. The rest of the device design is identical to that shown in Fig. 3.

**Fig. 6:**
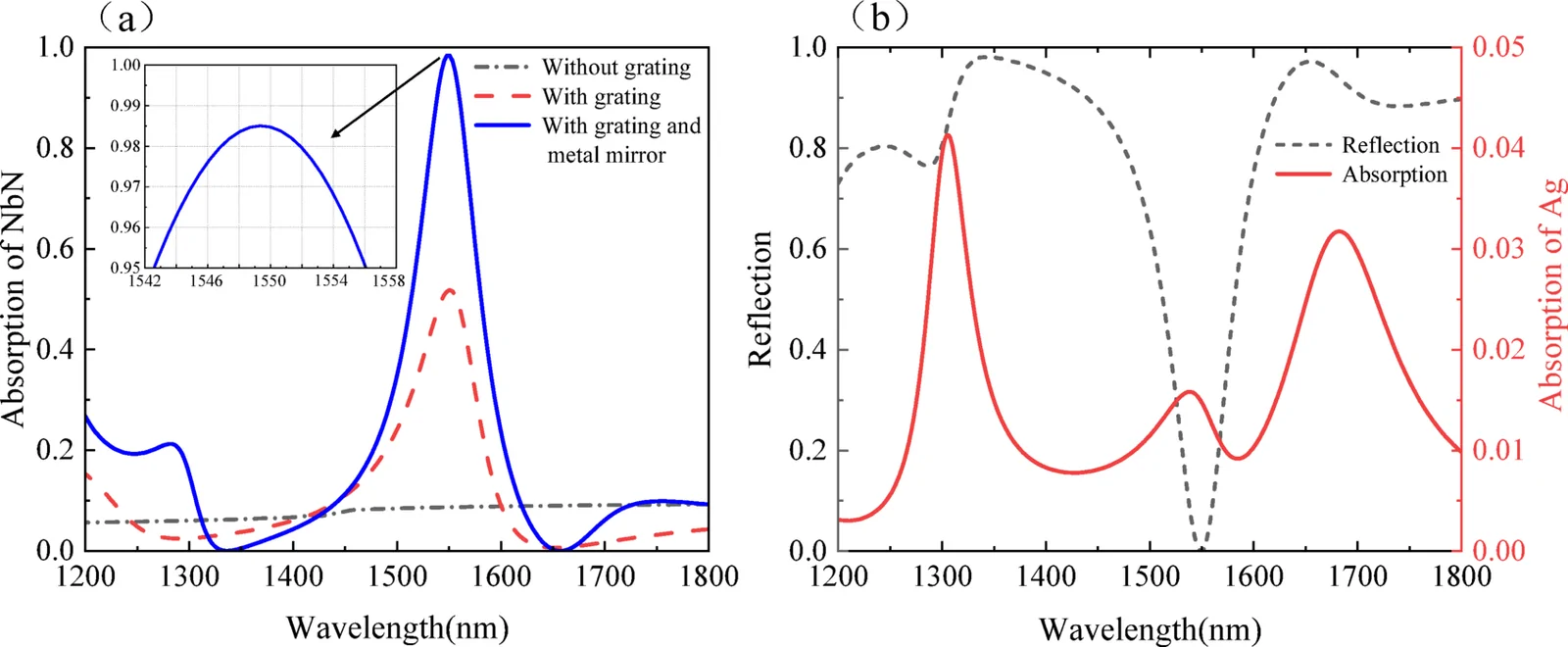
Optical response of the SNSPD structure with metallic mirror. (**a**) Absorption curves of NbN nanowires in the SNSPD without the grating, with the grating, and with both the grating and the metallic mirror. The inset shows an enlarged view of the absorption curves around λ = 1550 nm. (**b**) Reflection curve of the SNSPD and absorption curve of the metallic mirror (Ag).

The simulation still uses a 2D unit cell which is identical to that described in Fig. 4. Silver is added as one of the optical materials in the simulation, and its refractive index at 1550 nm is *n*_Ag_=0.39 + 10.17i^40^. In order to ensure that the absorption peak is located at λ = 1550 nm, we need to slightly adjust the width and thickness of the grating. The structural parameters used in the simulation are as follows: *w*_grating_=490 nm, *h*_grating_=510 nm, *d*_up_=50 nm, *d*_down_=150 nm, *w*_NbN_=100 nm, *d*_NbN_=7 nm and *p*=1000 nm. The simulation result is shown in Fig. 6(a) and compared with the results shown in Fig. 4(a).

Figure 6(a) shows that the absorption of the NbN nanowires is significantly enhanced by adding the silicon dielectric grating and metallic mirror to the device. At λ = 1550 nm, the absorption efficiency of the NbN nanowires can exceed 98% when the duty cycle of the NbN nanowires is only 10%. The inset shows an enlarged view of the absorption curve around λ = 1550 nm. Compared with SNSPDs employing optical cavity structures, this design can more effectively trap the incident light around the NbN nanowires through the Fano resonance excited by the dielectric grating. Therefore, the SNSPD can more easily meet the performance requirements for high-efficiency detection. In addition, since the duty cycle of the NbN nanowires is only 10%, the corresponding kinetic inductance of the nanowires is reduced, which effectively shortens the recovery time of the SNSPD. According to a previous study, the recovery time of SNSPDs is approximately 12 ns when the filling factor of the NbN nanowires is around 12%^21^. The SNSPD proposed in this work will exhibit faster recovery characteristics while maintaining the same active sensing area. Finally, the SNSPD can also meet the performance requirements for high-speed detection.

Considering that the absorption efficiency of the NbN nanowires is not 100% at λ = 1550 nm, the reflection curve of the SNSPD and the absorption curve of the metallic mirror (Ag) are calculated in Fig. 6(b). The results show that the incident light not absorbed by the NbN nanowires is mainly dissipated in the metallic mirror (~ 1.47%) at λ = 1550 nm. To further improve the absorption efficiency of the nanowires, reflective materials with lower loss need be adopted.

## Discussion

**Fig. 7:**
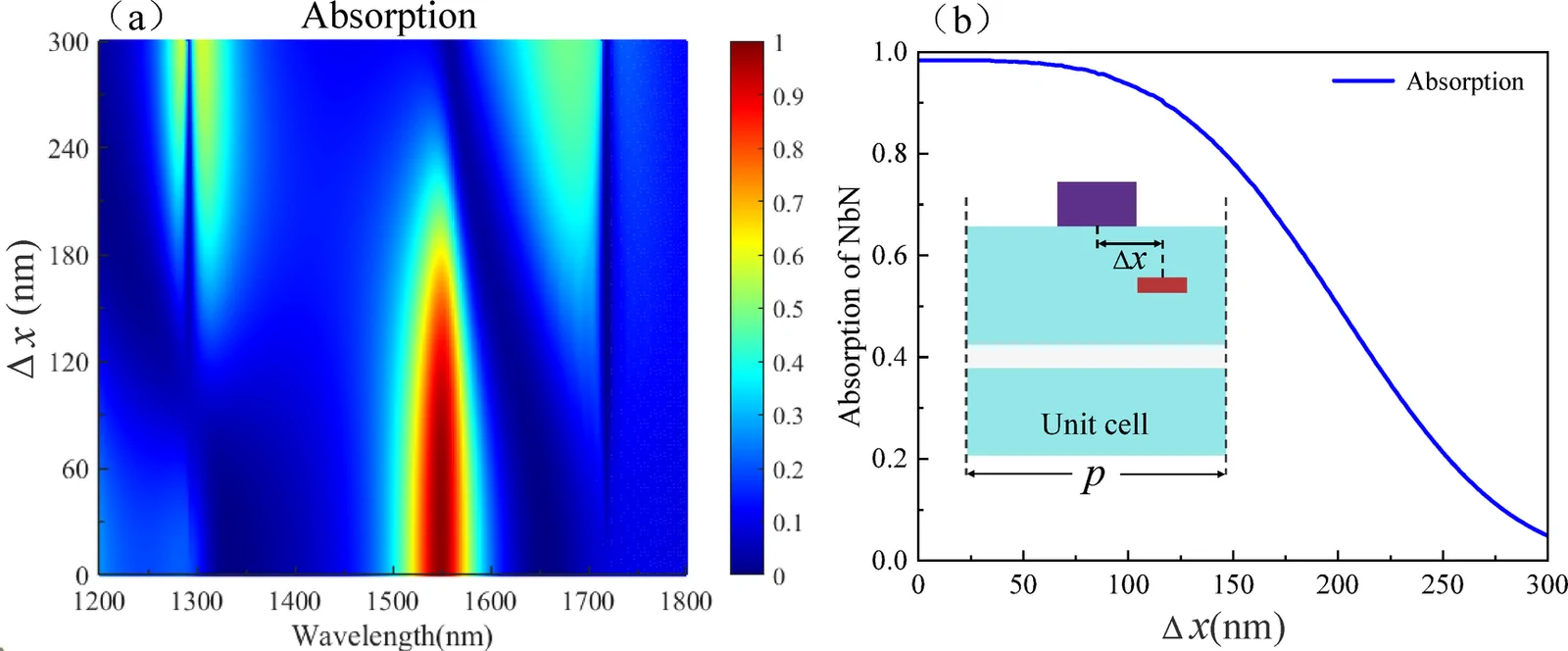
Effect of fabrication errors on the absorption efficiency of the SNSPD. (**a**) Absorptance spectrum as a function of Δ*x* for the wavelength ranging from 1200 nm to 1800 nm. (**b**) Absorption curve as a function of Δ*x* at λ = 1550 nm. The inset shows the schematic diagram of the structure under fabrication errors.

Considering the inevitable fabrication errors of SNSPDs during the preparation process, the position deviation between the nanowire and the grating is discussed in this work. The offset distance between the center of the nanowire and the center of the grating is defined as Δ*x*, which is shown in the inset of Fig. 7(b). The absorptance spectrum as a function of Δ*x* for the wavelength ranging from 1200 nm to 1800 nm is shown in Fig. 7(a). It can be seen that as Δ*x* increases from 0 to 180 nm, the absorption efficiency at λ = 1550 nm decreases accordingly, while the influence on the other wavelength is negligible. As Δ*x* increases further, the absorption efficiency at λ = 1550 nm continues to decrease, and the absorption efficiency near λ = 1300 nm and λ = 1700 nm gradually increases. Figure 7(b) shows the absorption curve as a function of Δ*x* at λ = 1550 nm. The absorption efficiency of the NbN nanowires gradually decreases with the increase of Δ*x*. However, the absorption efficiency still remains above 95% when Δ*x* ≤ 90 nm. It indicates that the proposed device exhibits excellent fabrication tolerance.

## Methods

The optical response of the proposed SNSPD structure was investigated using an optical simulation software based on finite-difference time-domain. All calculations were carried out on a personal computer equipped with an Intel(R) Core(TM) i3-13100 CPU and 16 GB RAM.

The complex dielectric function of NbN was described using a Drude model^39^. The model parameters were extracted from experimental measurements reported in Ref.^39^ and have been widely adopted in previous SNSPD simulations and design studies, such as the MIT, NIST and SIMIT^12,21,26,36,39,41^. Following these groups, the same Drude model was employed in this study. In addition, nonlinear optical effects in NbN were neglected, since the detector operates in the single-photon regime and the optical intensity is extremely low.

Silver was introduced in the optical design as a reflective efficient metal. In the simulation, its permittivity can be described using a dispersive model such as the Drude-Lorentz formulation to properly account for its wavelength-dependent response^40^. For the dielectric materials, the refractive indices were taken as *n*_sio2_=1.444 and *n*_si_=3.478 in this study, which were taken from Ref.^38^ and these values were commonly used in the design of SNSPDs.

## Conclusion

In summary, an ultralow-filling-factor SNSPD is designed using a one-dimensional silicon dielectric grating. The structure traps the incident optical energy near the NbN nanowires by Fano resonance, which is excited by the grating. Combining the metallic mirror, the optical absorption of the NbN nanowires is significantly enhanced. Numerical results show that the optical absorption of the NbN nanowires can exceed 98% at λ = 1550 nm under idealized structural conditions even when the nanowire filling factor is only 10%. Furthermore, the potential errors during the device fabrication are discussed. The absorption efficiency remains above 95% when the offset Δ*x* is less than 90 nm, demonstrating excellent fabrication tolerance. This work provides a potential design strategy for the future research on high-efficiency and high-speed SNSPDs.

## Supplementary Information

Below is the link to the electronic supplementary material.

Supplementary Material 1

Supplementary Material 2

Supplementary Material 3

Supplementary Material 4

Supplementary Material 5

## Data Availability

All data generated or analysed during this study are included in this published article and its supplementary information files.
