# Supplementary material for: Design of a Fano-resonance-enhanced dielectric grating for ultralow-filling-factor superconducting nanowire single-photon detector
Source: Sci Rep. 2026 Jun 20;16:28268. doi: 10.1038/s41598-026-58781-8 (PMC13558626; doi:10.1038/s41598-026-58781-8)
Supplement: Supplementary file 1 [file 41598_2026_58781_MOESM5_ESM.pdf]

Supplementary Information for

High-efficiency and ultralow-filling-factor superconducting nanowire single-photon detector enabled by Fano resonance

Fan Zheng, Kejing Wei, Xiuli Huang, Junxia Zhao, Nan Chen, Jing Qiang, Lili Cheng, Ang Li*

Supplementary Note 1. Simulation methodology

The optical response of the proposed superconducting nanowire single-photon detector (SNSPD) was investigated using a finite-difference time-domain (FDTD) method.

A two-dimensional unit cell model with periodic boundary conditions in the lateral direction and perfectly matched layers (PML) along the propagation direction was employed. The incident light was modeled as a plane wave propagating from air to the substrate, with the electric field polarized parallel to the nanowire.

The mesh size in the NbN nanowire region was set to 1 nm to accurately capture the near-field distribution, while a non-uniform mesh (1–50 nm) was applied in other regions to optimize computational efficiency.

Supplementary Note 2. Material parameters

The refractive indices used in the simulations at λ = 1550 nm are listed below:

Material	Refractive index
Si	n = 3.478
SiO_2	n = 1.444
NbN	n = 5.23 + 5.82i
Ag	n = 0.39 + 10.17i

The dielectric function of NbN was described using a Drude model based on literature values. The optical response of silver (Ag) was modeled using a Lorentz–Drude (LD) dispersion model to accurately describe its frequency-dependent permittivity. The model parameters were adopted from the well-established experimental data reported by Rakic et al. (Appl. Opt., 1998), ensuring reliable representation of the dispersive and absorptive properties of Ag in the near-infrared region.

Supplementary Note 3. Device structural parameters

Without metallic mirror (Fig. 3–4)

- Grating width: 475 nm

- Grating height: 500 nm
- Period: 1000 nm
- NbN width: 100 nm
- NbN thickness: 7 nm
- Upper SiO_2 layer: 50 nm

With metallic mirror (Fig. 5–6)

- Grating width: 490 nm
- Grating height: 510 nm
- Period: 1000 nm
- NbN width: 100 nm
- NbN thickness: 7 nm
- Upper SiO_2 layer: 50 nm
- Lower SiO_2 spacing: 150 nm
- Ag mirror thickness: 200 nm

Supplementary Note 4. Description of Supplementary Data

Supplementary Data 1: Absorption spectra

This dataset contains the simulated absorption efficiency of NbN nanowires corresponding to:

- Fig. 4(a): With and without grating
- Fig. 6(a): With grating and metallic mirror

File format: txt

Columns:

- Wavelength (nm)
- Absorption (without grating)
- Absorption (with grating)
- Absorption (with grating + mirror)

Supplementary Data 2: Reflection and transmission spectra

This dataset corresponds to Fig. 4(b), including:

- Reflection spectrum

- Transmission spectrum

File format: txt

Columns:

- Wavelength (nm)
- Reflection
- Transmission

Supplementary Data 3: Reflection and Absorption spectra of Ag

This dataset corresponds to Fig. 6(b), including:

- Reflection spectrum
- Absorption spectra of Ag

File format: txt

Columns:

- Wavelength (nm)
- Reflection
- Absorption of Ag layer

Supplementary Data 4: Fabrication tolerance analysis

This dataset corresponds to Fig. 7(b), showing the effect of nanowire-grating offset (Δx).

File format: txt

Columns:

- Offset Δx (nm)
- Absorption at 1550 nm

Supplementary Note 5. Additional remarks

All datasets were generated from numerical simulations under idealized structural conditions.
